# Mnemonic and attentional roles for states of attenuated alpha oscillations in perceptual working memory: a review

**DOI:** 10.1111/ejn.13759

**Published:** 2017-11-22

**Authors:** Freek van Ede

**Affiliations:** ^1^ Oxford Centre for Human Brain Activity Wellcome Centre for Integrative Neuroimaging Department of Psychiatry, University of Oxford Oxford UK

**Keywords:** attentional prioritisation, electroencephalography, neuronal oscillations, sensory recruitment, working memory retention

## Abstract

Alpha oscillations are often reported to be amplified during working memory (WM) retention, serving to disengage sensory areas to protect internal representations from external interference. At the same time, contemporary views of WM postulate that sensory areas may often also be recruited for retention. I here review recent evidence that during such ‘perceptual’ WM, alpha oscillations in mnemonically relevant sensory areas are not amplified but attenuated instead. I will argue that such attenuated alpha states serve a mnemonic role and, further, that larger attenuation may support item‐specific attentional prioritisation within perceptual WM. In critically evaluating this role, I also consider (and argue against) four alternatives to a strictly mnemonic account of the available data that may also prove useful to consider in future research. Finally, I highlight key implications of these data for the study of WM and for our understanding of the functional roles of states of attenuated alpha oscillations in cognition.

## Introduction

Alpha oscillations (8–12 Hz) provide a window into the engagement of the underlying neural circuity within macroscopic magneto‐ and encephalography (M/EEG) measurements in healthy humans (Berger, 1929; Hari & Salmelin, 1997) – with states of attenuated (vs. amplified) alpha oscillations generally being associated with increased (vs. decreased) cortical engagement (Klimesch *et al*., 2007; Jensen & Mazaheri, 2010; Foxe & Snyder, 2011; Haegens *et al*., 2011a; Hanslmayr *et al*., 2012). Because alpha oscillations are particularly well characterised in the brain's visual, somatosensory and auditory processing areas (e.g. Hari & Salmelin, 1997; Haegens *et al*., 2015), this role has been particularly well documented in relation to the gating of sensory information in service of adaptive perception.

In the realm of working memory (WM), alpha oscillations are typically reported to be amplified during retention (Klimesch *et al*., 1999; Jensen *et al*., 2002; Tuladhar *et al*., 2007; Haegens *et al*., 2010; Johnson *et al*., 2011; Bonnefond & Jensen, 2012; Obleser *et al*., 2012; Spitzer & Blankenburg, 2012), with larger increases in amplitude (e.g. Jensen *et al*., 2002; Tuladhar *et al*., 2007) as well as interareal and cross‐frequency phase synchronisation (Palva *et al*., 2010; Siebenhühner *et al*., 2016) with higher WM loads. There is growing consensus (Roux & Uhlhaas, 2014; Payne & Sekuler, 2014 for reviews; but see Palva & Palva, 2007) that these modulations too may reflect sensory gating, whereby internal representations are protected from (potential) interfering external sensory input through disengagement of the areas that are receptive to this input. In support of this, the amplification of posterior alpha oscillations during WM retention is more pronounced when prospective visual distractors are expected to cause larger interference (Bonnefond & Jensen, 2012), and similar distractor dependence has recently been documented in the auditory domain (Wöstmann *et al*., 2017).

In a traditional view of WM, in which representations are retained predominantly outside the sensory areas (as discussed in e.g. Sreenivasan *et al*., 2014), sensory disengagement appears a sensible and powerful protective instrument. Yet, increasing evidence suggests that WM retention may often also recruit sensory areas (e.g. Awh & Jonides, 2001; Pasternak & Greenlee, 2005; Harrison & Tong, 2009; Kuo *et al*., 2009; Serences *et al*., 2009; Sreenivasan *et al*., 2014; D'Esposito & Postle, 2015), particularly when the fine‐grained sensory properties of the memoranda are retained (Lee *et al*., 2013; Christophel *et al*., 2017). To reconcile sensory recruitment during such ‘perceptual’ WM with the postulated alpha‐mediated sensory disengagement, we are invited to revisit the link between alpha oscillations and WM in at least two ways.

First, the utility of alpha amplification for distractor inhibition may be largely restricted to circumstances in which non‐sensory properties (e.g. semantic category labels) are retained, or in which retained memoranda engage a different sensory brain areas than the area from which alpha oscillations are considered. Indeed, amplification of posterior alpha oscillations appears particularly prominent in tasks where the content of WM is verbal, or somatosensory (Jensen *et al*., 2002; Haegens *et al*., 2010; Bonnefond & Jensen, 2012; see also Gevins *et al*., 1997; but see Johnson *et al*., 2011), but where the sources of this amplification localise to visual brain areas (Tuladhar *et al*., 2007; Haegens *et al*., 2010; Bonnefond & Jensen, 2012). Second, it raises the possibility that modulations of alpha oscillations during WM may also serve a genuine mnemonic function – whereby attenuated alpha states (marking sensory engagement, or ‘recruitment’) actively support WM retention.

Building on earlier reviews on the role of alpha oscillations in WM that focused and converged on a protective (disengaging) role for states of amplified alpha oscillations during WM (Payne & Sekuler, 2014; Roux & Uhlhaas, 2014), I here review recent evidence for this complementary mnemonic role for states of attenuated alpha oscillations during perceptual WM. I first highlight recent evidence showing that, when mnemonic information is relevant to a particular sensory area, alpha oscillations measured from this area are not amplified but attenuated instead. In addition, I discuss recent evidence that such attenuated alpha states may not only support WM retention but may also support attentional prioritisation within WM and govern item accessibility. I finally consider (and, where possible, counter) four alternative interpretations of the available data that challenge a strictly mnemonic interpretation and highlight key implications of the reviewed work.

## A mnemonic role for attenuated alpha states in perceptual working memory

A number of recent studies have demonstrated that alpha oscillations are not only modulated in sensory modalities that are irrelevant to the WM retention (e.g. in visual areas during verbal retention; as in Jensen *et al*., 2002; Tuladhar *et al*., 2007; Bonnefond & Jensen, 2012) but also in modalities that are considered relevant for retention. For example, on the basis of the popular lateralised visual change‐detection task (as in Vogel & Machizawa, 2004), several studies have now demonstrated that posterior (visual) alpha oscillations show a corresponding lateralisation that sustains throughout the retention interval – whereby alpha amplitude is lower contralateral vs. ipsilateral to the retinotopic location of the retained items at encoding (Grimault *et al*., 2009; Sauseng *et al*., 2009; Lozano‐Soldevilla *et al*., 2014). In a similar vein, studies have reported a relative attenuation of alpha in mnemonically relevant vs. mnemonically irrelevant sensory modalities (Spitzer & Blankenburg, 2012; van Ede *et al*., 2017a), as well as when retaining spatial location as opposed to visual identity (Jokisch & Jensen, 2007), temporal order (Roberts *et al*., 2013) or relational (Ikkai *et al*., 2014) information.

Still, as the core results in these studies typically entail a relative amplitude difference between mnemonically relevant vs. irrelevant areas, it often remains difficult to distinguish cortical engagement by attenuated alpha oscillations in relevant areas (supporting mnemonic retention) from cortical disengagement by amplified alpha oscillations in irrelevant areas (supporting distractor inhibition). Complementary evidence suggests that both scenarios may be at play. For example, in the lateralised change‐detection task, alpha lateralisation appears particularly sensitive to the number of irrelevant items (Sauseng *et al*., 2009) suggesting a role in distractor inhibition. At the same time, in the context of a related lateralised visual WM task, performance appears best predicted by alpha states in visual sites contralateral to the probed item's retained location (with lower amplitude predicting better performance; van Ede *et al*., 2017b; see also Spitzer & Blankenburg, 2012), thus arguing for a role in mnemonic retention.

To further disentangle these possibilities, my colleagues and I recently investigated alpha modulations in both visual and somatosensory brain areas during retention of either visual or somatosensory sequences. In addition to directly comparing visual with somatosensory retention, we also compared each separately to a control condition with no retention demand (i.e. load 0). This yielded an unambiguous pattern of results: during retention of visual sequences, alpha (as well as beta) oscillations were almost exclusively attenuated in visual brain areas, whereas during retention of somatosensory sequences, this was the case in somatosensory brain areas (van Ede *et al*., 2017a). In both modalities, these WM related attenuations of alpha and beta oscillations scaled with load (see also Fukuda *et al*., 2015; Erickson *et al*., 2017). These results could not simply be accounted for by differences in sensory processing, as sensory stimulation was equated between control and WM trials, between visual and somatosensory WM trials and between trials with different WM loads (van Ede *et al*., 2017a, for details).

**Figure 1:**
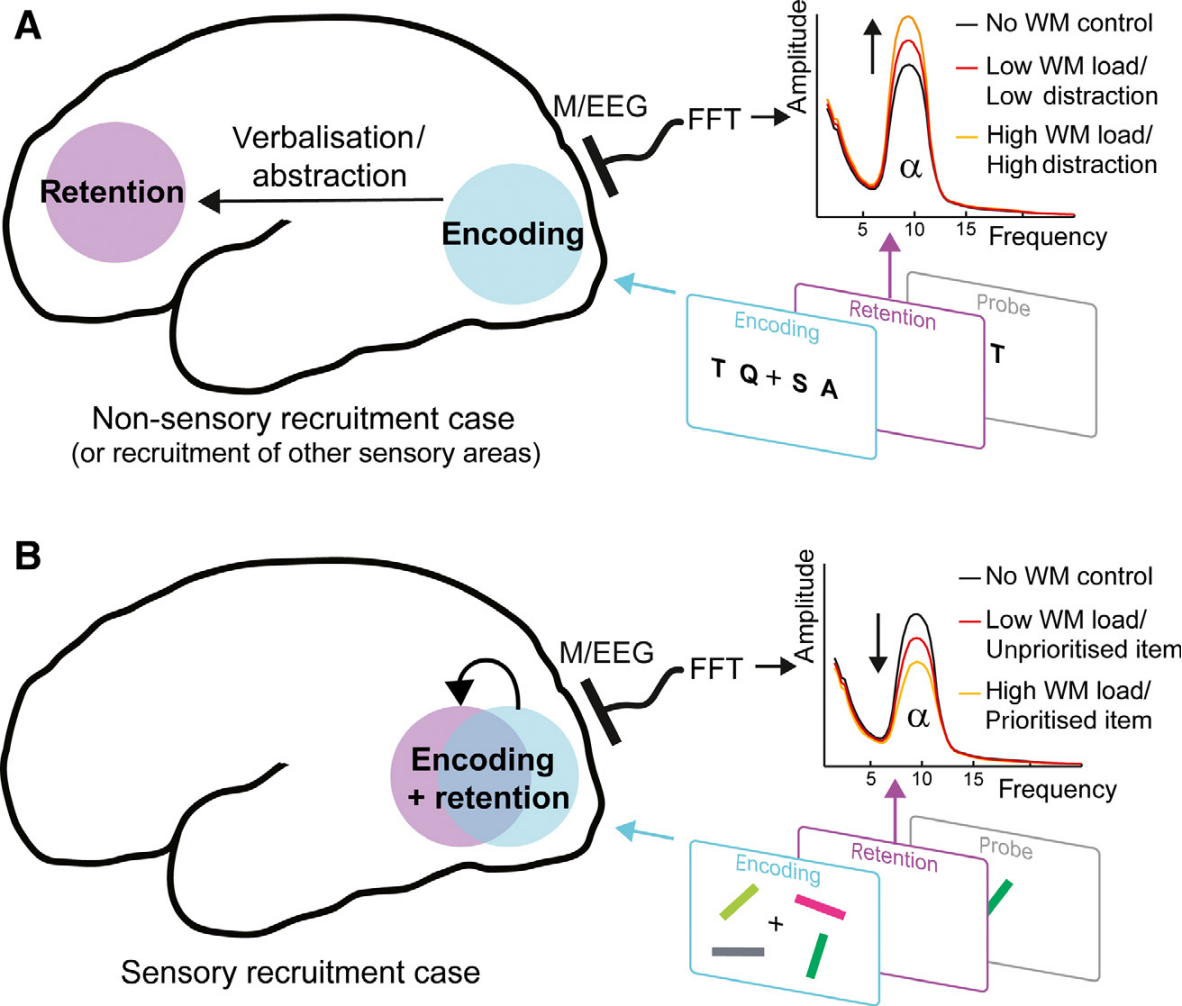
Alpha amplitude during working memory retention as a function of sensory recruitment, external distraction and item‐specific prioritisation. In scenario (A), the memory array contains verbal material that is encoded in visual areas but retained elsewhere (e.g. in prefrontal, or language areas). In M/EEG recordings from the visual areas during the retention interval, alpha power increases with load (as in e.g. Jensen *et al*., 2002), as well as with the level of (anticipated) external distraction (as in e.g. Bonnefond & Jensen, 2012). In contrast, in scenario (B), the memory array contains visual items that, this time, are not only encoded but also retained in visual areas (because the task requires the retention of the precise visual identity – orientations and colours – of the items). Alpha power in M/EEG recordings from the visual areas now decreases with load (as in e.g. van Ede *et al*., 2017a) and decreases further when items are placed in a prioritised state based on current attentional demands (as in e.g. Myers *et al*., 2015). How (expected) distraction in the sensory recruitment case (scenario B) and item‐specific prioritisation in the non‐sensory recruitment case (scenario A) affect alpha remains to be more systematically investigated and is therefore not included in the schematic. M/EEG, Magneto/Electroencephalography; FFT, fast Fourier transform.

As schematically depicted in Fig. 1, when directly comparing the results of this study to those in the landmark study by Jensen *et al*. (2002), an important insight emerges. In both cases, alpha amplitude during WM retention shows prominent modulation by WM load. However, the sign of these modulations is opposite. When participants engage in verbal retention (even when information is encoded visually, as in Jensen *et al*., 2002; Tuladhar *et al*., 2007), the amplitude of posterior (putatively visual) alpha increases with load, whereas when they engage in visual retention (as argued in van Ede *et al*., 2017a), alpha amplitude now decreases with load (for more demonstrations of alpha attenuation during WM see also: Medendorp *et al*., 2007; Fukuda *et al*., 2015; Erickson *et al*., 2017). The direction of modulation thus appears to be highly dependent on the nature of the memoranda (as well as the source of the alpha oscillations under consideration; as in e.g. Spitzer & Blankenburg, 2012; van Ede *et al*., 2017a).

Several recent studies have further demonstrated a link between alpha oscillations during retention and the content‐specific identities of WM representations using sophisticated forward encoding modelling of visual stimulus features such as location and orientation (e.g. Foster *et al*., 2016; Fukuda *et al*., 2016). This work too argues for a mnemonic role for alpha oscillations during perceptual WM and shows that this role extends beyond the modality and spatial location of the mnemonic items to also include their feature‐specific identity (Fukuda *et al*., 2016). It remains to be evaluated, however, precisely what aspects of the alpha oscillations (spatial patterns of attenuation, amplification, or both, as well as orthogonal aspects such as changes in peak frequency) contribute to the reconstruction of the memorised stimulus features.

## Attenuated alpha states reflect behaviourally relevant prioritisation within perceptual working memory

Over the past decade, it has become clear that attentional selection and prioritisation continue to operate on perceptual representations, even after they have been encoded into WM (Oberauer, 2002; Griffin & Nobre, 2003; Landman *et al*., 2003; Zokaei *et al*., 2014; Souza & Oberauer, 2016; Myers *et al*., 2017). Ample evidence in the perceptual domain suggests that modulations of alpha oscillations may play a key role in such attentional processes (Foxe *et al*., 1998; Worden *et al*., 2000; Thut *et al*., 2006; Jones *et al*., 2010; van Ede *et al*., 2011, 2012; Haegens *et al*., 2011b), with lower amplitude of relevant sensory areas predicting better perceptual performance (e.g. van Dijk *et al*., 2008; Jones *et al*., 2010; van Ede *et al*., 2012). This raises the possibility that, in addition to mnemonic retention, attenuated alpha states may also play a role in mnemonic prioritisation and, accordingly, impact mnemonic performance (at least in cases of perceptual WM).

Support for this hypothesis was recently provided by a number of related studies. The majority of these studies investigated this using retro‐cues (i.e. cues presented during WM retention) that inform which item will (most likely) be probed after an additional delay (see Souza & Oberauer, 2016 for a review of retro‐cueing). As for the lateralised change‐detection tasks described above (where attentional selection occurs during encoding), these studies all report similar lateralisation of alpha oscillations relative to the original location of the retro‐cued item (Spitzer & Blankenburg, 2011; Poch *et al*., 2014; Myers *et al*., 2015; Schneider *et al*., 2015; Wallis *et al*., 2015; Mok *et al*., 2016; Wolff *et al*., 2017). These lateralised alpha modulations do not, however, appear to be exclusive to situations in which mnemonic prioritisation is directed by retro‐cues. They can also be dynamically steered by internally guided temporal expectations, when prioritisation is directed by subjective estimates of elapsed time (van Ede *et al*., 2017b), or by the expected order in which items will become relevant for visual search (de Vries *et al*., 2017).

Prioritisation in WM is known to have a profound influence on performance (Souza & Oberauer, 2016 for review). If states of attenuated alpha oscillations indeed support such mnemonic prioritisation (with larger attenuation in populations retaining the prioritised item; as also indicated in Fig. 1), then one would expect such states (at the time of probing) to be predictive of performance on a trial‐by‐trial basis. My colleagues and I recently reported precisely this (van Ede *et al*., 2017b; see also Poliakov *et al*., 2014; Backer *et al*., 2015). In our data, this predictive influence (lower amplitude predicting faster WM access times upon probing) was particularly prominent in sites contralateral to the location of the probed item at encoding (despite the fact that the probe was always presented centrally) and to the interval immediately preceding the probe. Moreover, this influence was particularly prominent on item accessibility, or decision time (as was the influence of our attentional manipulation), and we have now replicated this effect (F. van Ede, S. Chekroud, AC. Nobre, unpublished data). Together, these data suggest that, even when several mnemonic items can all be recalled with high precision, the immediate availability of these items is variable and appears to be tracked by the degree of alpha attenuation in a spatiotopically preserved manner.

Finally, it is noteworthy that the vast majority of studies linking attention to WM (as well as alpha to attention) regard spatial‐based prioritisation mechanisms. It has recently been shown that visual feature dimensions (such as orientation and colour of all items in the array) can also be dynamically up‐ and downregulated during visual WM retention (Ye *et al*., 2016; Niklaus *et al*., 2017). Whether alpha modulations also support such feature dimension‐based attentional prioritisation (as reported for the perceptual domain; see Snyder & Foxe, 2010), remains an interesting target for future research.

## Alternative accounts to mnemonic retention and prioritisation

While it is tempting to attribute the reviewed work on alpha modulations during WM retention intervals to a mnemonic role, I will argue that such ‘temporal localisation’ to the retention interval is in itself not sufficient to warrant this inference. There are at least four alternative accounts that need to be considered. I go over these below and highlight the available counter‐evidence for each. This serves not only to rule out these alternatives (although there is probably not a single study that can compellingly counter all of them) but also to outline relevant considerations when designing and interpreting future experiments that target mnemonic and attentional roles of neural activity during WM retention.

### Mnemonic retention vs. probe anticipation

Ample evidence points to a key role for modulations of alpha oscillations in anticipatory attention of upcoming perceptual events (e.g. Foxe *et al*., 1998; Worden *et al*., 2000; Thut *et al*., 2006; Jones *et al*., 2010; van Ede *et al*., 2011, 2012; Haegens *et al*., 2011b). As WM retention periods are often followed by a perceptual probe stimulus (or a probe display containing multiple items), alpha modulations observed during retention need not reflect retention per se but may reflect probe anticipation instead. This is particularly a concern in tasks in which mnemonic items are probed at their encoded locations (and/or modality), and in which probe displays also scale with load (as is typically the case in the popular visual change‐detection task; as described in Luck & Vogel, 1997; Vogel & Machizawa, 2004). Although probe anticipation may thus account for a fair share of all reports of alpha modulations during WM retention, not all documented alpha modulations during retention can be explained by probe anticipation. For example, alpha modulations scale with load, even when the probe displays are independent of load (as in Jensen *et al*., 2002; van Ede *et al*., 2017a) and appear highly specific to the modality of the retained memoranda (e.g. visual vs. somatosensory), even if probe displays are always visual (van Ede *et al*., 2017a). Also, the lateralisation (relative to the side of the mnemonic items at encoding) of alpha oscillations during WM retention does not appear to rely on lateralised probe anticipation, as this is observed even when items are probed centrally through their colour (van Ede *et al*., 2017b).

### Mnemonic retention vs. lingering of sensory encoding or retro‐cue processing

In the opposite direction of probe anticipation, one must also consider potential lingering of neural responses related to perceptual encoding – as well as to processes of attentional selection during encoding. It is well known that sensory input itself also attenuates alpha oscillations (possibly followed by a rebound) and that such state changes can linger for at least up to a second after this input (e.g. Hari & Salmelin, 1997; Cheyne *et al*., 2003). Such lingering of encoding responses into the retention interval is particularly problematic when sensory input is not well matched between conditions (such as when different load conditions have different number of items at encoding, or when items are only presented in one sensory modality, but when retention activity is compared between modalities). However, even when sensory input is matched, one is still confronted with potential lingering of the attentional selection (of the mnemonically relevant items) during encoding. There are several arguments against this potential alternative account (in addition to the observation that alpha modulations appear to sustain throughout retention intervals of three or more seconds; e.g. Jensen *et al*., 2002; van Ede *et al*., 2017a). Foremost, as reviewed above, alpha modulations also occur following retro‐cues that enable selection of the relevant item(s) only after encoding (but see below). Moreover, it has recently been observed that lateralised modulations of alpha during retention are sensitive to attentional switches between items that occur more than one‐second after encoding and that are guided solely by internal estimates of elapsed time (van Ede *et al*., 2017b). Finally, if alpha states during retention reflected mere lingering from (attentional selection during) encoding, then, intuitively, mnemonic performance would be best predicted by alpha amplitude during and immediately after encoding. In contrast, this relation appears particularly pronounced immediately prior to the probe (van Ede *et al*., 2017b; but see Poliakov *et al*., 2014).

Building on the sensory encoding case, lateralised alpha modulations following retro‐cues may sometimes relate to processing of the retro‐cue itself, as opposed to reflecting shifts in the focus of attention during WM retention. However, the attentional lateralisation of alpha during WM retention appears specific to the modality and location of the retained memoranda, even when the retro‐cue is presented in another modality and does not contain any spatial information (i.e. Spitzer & Blankenburg, 2011) and occurs even when attention is directed via internally guided temporal expectations as opposed to retro‐cues (van Ede *et al*., 2017b; de Vries *et al*., 2017).

### Mnemonic retention vs. oculomotor behaviour

As was already discovered by Hans Berger, posterior alpha oscillations are profoundly amplified by closing one's eyes (Berger, 1929). This immediately raises the question whether posterior alpha modulations during WM retention may merely reflect modulations in the degree to which the eyes (or pupils) are open. Indeed, closing one's eyes might be another effective (but from a cognitive neuroscience point of view less interesting) way of blocking out potential distracting visual input. If so, these modulations would be expected to be largely confined to posterior alpha oscillations. In contrast, alpha modulations have been described in multiple sensory modalities, where they appear to be selectively modulated depending on the nature of the retained memoranda (e.g. Spitzer & Blankenburg, 2012; van Ede *et al*., 2017a; Wöstmann *et al*., 2017). Moreover, potential changes in eye‐opening and pupil size cannot account for lateralised patterns of alpha oscillations relative to the spatiotopic location of the retained and prioritised memoranda (e.g. Sauseng *et al*., 2009; Spitzer & Blankenburg, 2011; Myers *et al*., 2015). In the latter case, however, mnemonically driven gaze shifts are a concern instead. Indeed, even when there may be nothing to look at after encoding, participants may still engage in gaze shifts to and between items’ encoded locations (Ferreira *et al*., 2008; Williams *et al*., 2013). However, correcting for such gaze shifts appears to preserve alpha lateralisation during WM retention (van Ede *et al*., 2017b). Moreover, gaze shifts cannot easily account for alpha lateralisation in somatosensory sites during somatosensory WM (Spitzer & Blankenburg, 2011).

### Mnemonic retention vs. WM guided interactions with incoming sensations

Although laboratory studies of WM often aim to minimise sensory input during WM retention (such as by having people stare at a blank screen while retaining visual items in WM), it is also evident that it is not possible to deprive participants from sensory input altogether. This raises the possibility that modulations of alpha oscillations during WM retention reflect some interaction between the current content of WM and the (residual) sensory input registered during the retention interval (i.e. WM guided interactions with incoming sensations). In situations in which incoming sensory input can serve WM retention (e.g. the border of a computer monitor may provide a useful spatial reference frame), alpha may attenuate as a result of attentively processing this input. It is debatable, however, whether this constitutes a genuine alternative to a mnemonic interpretation. If we consider that mnemonic retention of perceptual representations may inherently entail a ‘projection’ of these representations onto ongoing perceptual streams (which will be particularly pertinent in the rich sensory environments encountered in everyday situations), then we may wish to attribute the neural signatures of this interaction to WM as well. Still, the degree to which such interactions between WM representations and incoming sensations shape alpha modulations during retention remains a largely unexplored territory (although some first important steps are being made; e.g. de Vries *et al*., 2017).

## Implications and future directions

### Alpha oscillations and cognition

On face value, attenuated alpha states in mnemonically relevant sensory areas may appear simply as the flipside of amplified alpha states in mnemonically irrelevant sensory areas. However, whereas the latter may still be attributed to a sensory gating function (albeit in the context of WM), this is not the case for the former. As I have argued, such states of attenuated alpha oscillations in early sensory areas may serve a genuine mnemonic function instead – as has also been argued in the context of long‐term memory retrieval (e.g. Hanslmayr *et al*., 2012; Staresina *et al*., 2016; Waldhauser *et al*., 2016). This bears implications for our understanding of the functional role of attenuated alpha states in cognition. For example, it has recently been argued that such states predominantly reflect an ‘excitability bias’ that merely increases the likelihood of perceiving sensations amidst a noisy background and that may even lead to non‐veridical percepts (Lange *et al*., 2013; Iemi *et al*., 2017). In contrast, the reviewed work suggests that such states are also functionally relevant for WM, in cases where the items are clearly registered at encoding. This is the case not only for attenuated alpha states prior to WM encoding (Myers *et al*., 2014), but, as reviewed throughout, also for such states during the ensuing retention period. Beyond mere excitability biases with a predominant sensory gating function, such states in sensory areas may thus also serve mnemonically relevant computations (I have focused on retention and prioritisation, but this may possibly also extend to computations such as refreshing, updating and manipulating) on previously encoded perceptual representations.

A key open question regards the precise nature of the neural computations associated with (or enabled by) states of attenuated alpha oscillations (as well as their means of carrying over into measurable modulations in extracranial M/EEG). While the answer to this important question remains largely unknown, it is worth pointing out that these neural computations are unlikely to carry only a mnemonic function. Indeed, it is well known that sensory processing also attenuates alpha oscillations and that such attenuation can also be instantiated during the mere anticipation of upcoming processing demands (e.g. Foxe *et al*., 1998; Worden *et al*., 2000; Thut *et al*., 2006; Jones *et al*., 2010; van Ede *et al*., 2011, 2012; Haegens *et al*., 2011b). It is thus likely that, rather than ‘coding’ for the items in WM themselves, such states enable neural computations that facilitate information processing, be it in the context of perception, action, WM, and so on. Influential models posit that alpha oscillations may be the consequence of rhythmic pulses of inhibition in which individual cycles contain only relatively short ‘windows of opportunity’ (when inhibition dies off) for neurons to fire and exchange information (Klimesch *et al*., 2007; Jensen & Mazaheri, 2010; Jensen *et al*., 2014; Gips *et al*., 2016). If so, their attenuation may reflect reduced inhibitory pulsing, creating longer windows of opportunity and enhancing (the capacity for) information processing and transmission. Another, potentially complementary, possibility is that attenuated alpha states are associated with the decorrelation (desynchronisation) of neuronal firing rates (potentially through the segregation into multiple alpha subnetworks at a finer spatial scale), which may increase the coding capacity of the corresponding neuronal population (Zohary *et al*., 1994; Hanslmayr *et al*., 2012). Clearly, more work is needed to investigate these possibilities and how they relate to mnemonic as well as other cognitive processes and neural computations.

In future studies, it will also be informative to assess whether states of attenuated (or amplified) alpha oscillations causally contribute to WM retention and prioritisation. This could be achieved by experimentally manipulating these states through, for example, brain stimulation techniques (see Thut & Miniussi, 2009; Thut *et al*., 2012) and evaluating whether and how this impairs or facilitates memory performance (depending on the location and modality of the mnemonic items as well as the stimulation site, timing and frequency).

### Working memory and distractibility

Provided that states of attenuated alpha oscillations have traditionally been associated with increased susceptibility to incoming sensations, the question arises how potentially distracting sensory input is countered in situations in which WM retention involves attenuation of alpha oscillations. This is particularly pertinent for distractors that occupy the same modality and location as the retained memoranda. One implication of the reviewed work may be that the high perceptual resolution gained from retaining items in early sensory areas (putatively through attenuated alpha states) inherently comes at the cost of increased susceptibility to sensory interference from similar material (in line with e.g. Sreenivasan & Jha, 2007; Chumbley *et al*., 2008; Rademaker *et al*., 2015). At the same time, the fact that distractors that are perceptually more similar to retained WM representations lead to more interference does not mean that such distraction cannot be overcome at all. Indeed, there is evidence that perceptually similar items may sometimes even receive more inhibition than less similar items (Sreenivasan & Jha, 2007; Kiyonaga & Egner, 2016). Whether and how alpha modulations during retention can support this remains to be investigated and will depend a lot on the degree of spatial and feature specificity with which alpha can be modulated within mnemonically relevant sensory areas.

Another, and perhaps less intuitive, prediction that follows is that even placing an item in the focus of attention (i.e. prioritising an item) may come at the cost of increased susceptibility of this item to sensory interference (for which there is some evidence: Hu *et al*., 2014; although counter‐evidence is documented too: Makovski *et al*., 2008; Souza *et al*., 2016). In future research, it will be interesting to evaluate the extent to which such increased susceptibility to interference is associated with the extent to which alpha oscillations are attenuated (vs. amplified) in the mnemonically relevant sensory areas. It will further be interesting to investigate circumstances under which participants may trade in their high‐fidelity perceptual representations to more abstract representations (putatively gaining distractor resilience at the cost of perceptual resolution; see Lee *et al*., 2013; Christophel *et al*., 2017) and to see whether alpha oscillations track such transitions.

## Conclusion

I have argued that the association between alpha oscillations and WM is critically shaped by the nature of the memoranda, in conjunction with the source of the alpha oscillations under consideration. When a particular sensory area is not engaged for WM retention, alpha oscillations in this area may be amplified to suppress sensory interference. In contrast, when WM requires the retention of the fine‐grained perceptual representation and engages a particular sensory area, alpha oscillations in this area are attenuated instead. Focusing on the latter case, I have argued that such states of attenuated alpha oscillations serve a genuine and behaviourally relevant mnemonic function. Thus, in addition to a role for such ‘sensory states’ in gating incoming sensations, such states may also support neural computations associated with active retention and dynamic prioritisation in memory. At the same time, I have raised (and where possible countered) four alternatives to a strictly mnemonic account of the available data that, I hope, will also prove useful to consider in designing and interpreting future research.

## Conflict of Interest

The author has no conflict of interest.

## Author contributions

FvE researched the data and wrote the review.

## References

[ejn13759-bib-0001] Awh, E. & Jonides, J. (2001) Overlapping mechanisms of attention and spatial working memory. Trends Cogn. Sci., 5, 119–126. 11239812 10.1016/s1364-6613(00)01593-x

[ejn13759-bib-0002] Backer, K.C. , Binns, M.A. & Alain, C. (2015) Neural dynamics underlying attentional orienting to auditory representations in short‐term memory. J. Neurosci., 35, 1307–1318. 25609643 10.1523/JNEUROSCI.1487-14.2015 PMC6605545

[ejn13759-bib-0003] Berger, H. (1929) Über das elektroenkephalogramm des menschen. Arch. Psychiat. Nerven., 87, 527–570.

[ejn13759-bib-0004] Bonnefond, M. & Jensen, O. (2012) Alpha oscillations serve to protect working memory maintenance against anticipated distracters. Curr. Biol., 22, 1969–1974. 23041197 10.1016/j.cub.2012.08.029

[ejn13759-bib-0005] Cheyne, D. , Gaetz, W. , Garnero, L. , Lachaux, J.P. , Ducorps, A. , Schwartz, D. & Varela, F.J. (2003) Neuromagnetic imaging of cortical oscillations accompanying tactile stimulation. Brain Res. Cogn. Brain Res., 17, 599–611. 14561448 10.1016/s0926-6410(03)00173-3

[ejn13759-bib-0006] Christophel, T.B. , Klink, P.C. , Spitzer, B. , Roelfsema, P.R. & Haynes, J.D. (2017) The distributed nature of working memory. Trends Cogn. Sci., 21, 111–124. 28063661 10.1016/j.tics.2016.12.007

[ejn13759-bib-0007] Chumbley, J.R. , Dolan, R.J. & Friston, K.J. (2008) Attractor models of working memory and their modulation by reward. Biol. Cybern., 98, 11–18. 18080131 10.1007/s00422-007-0202-0 PMC2642585

[ejn13759-bib-0008] D'Esposito, M. & Postle, B.R. (2015) The cognitive neuroscience of working memory. Annu. Rev. Psychol., 66, 115–142. 25251486 10.1146/annurev-psych-010814-015031 PMC4374359

[ejn13759-bib-0009] van Dijk, H. , Schoffelen, J.M. , Oostenveld, R. & Jensen, O. (2008) Prestimulus oscillatory activity in the alpha band predicts visual discrimination ability. J. Neurosci., 28, 1816–1823. 18287498 10.1523/JNEUROSCI.1853-07.2008 PMC6671447

[ejn13759-bib-0010] van Ede, F. , de Lange, F. , Jensen, O. & Maris, E. (2011) Orienting attention to an upcoming tactile event involves a spatially and temporally specific modulation of sensorimotor alpha‐ and beta‐band oscillations. J. Neurosci., 31, 2016–2024. 21307240 10.1523/JNEUROSCI.5630-10.2011 PMC6633042

[ejn13759-bib-0011] van Ede, F. , Köster, M. & Maris, E. (2012) Beyond establishing involvement: quantifying the contribution of anticipatory α‐ and β‐band suppression to perceptual improvement with attention. J. Neurophysiol., 108, 2352–2362. 22896721 10.1152/jn.00347.2012

[ejn13759-bib-0012] van Ede, F. , Jensen, O. & Maris, E. (2017a) Supramodal theta, gamma, and sustained fields predict modality‐specific modulations of alpha and beta oscillations during visual and tactile working memory. J. Cognitive Neurosci., 29, 1455–1472. 10.1162/jocn_a_01129 28358658

[ejn13759-bib-0013] van Ede, F. , Niklaus, M. & Nobre, A.C. (2017b) Temporal expectations guide dynamic prioritization in visual working memory through attenuated alpha oscillations. J. Neurosci., 37, 437–445. 28077721 10.1523/JNEUROSCI.2272-16.2016 PMC5242399

[ejn13759-bib-0014] Erickson, M.A. , Albrecht, M.A. , Robinson, B. , Luck, S.J. & Gold, J.M. (2017) Impaired suppression of delay‐period alpha and beta is associated with impaired working memory in schizophrenia. Biol. Psychiat., 2, 272–279. 10.1016/j.bpsc.2016.09.003 PMC5487003 28670630

[ejn13759-bib-0015] Ferreira, F. , Apel, J. & Henderson, J.M. (2008) Taking a new look at looking at nothing. Trends Cogn. Sci., 12, 405–410. 18805041 10.1016/j.tics.2008.07.007

[ejn13759-bib-0016] Foster, J.J. , Sutterer, D.W. , Serences, J.T. , Vogel, E.K. & Awh, E. (2016) The topography of alpha‐band activity tracks the content of spatial working memory. J. Neurophysiol., 115, 168–177. 26467522 10.1152/jn.00860.2015 PMC4760461

[ejn13759-bib-0017] Foxe, J.J. & Snyder, A.C. (2011) The role of alpha‐band brain oscillations as a sensory suppression mechanism during selective attention. Front. Psychol., 2, 154. 21779269 10.3389/fpsyg.2011.00154 PMC3132683

[ejn13759-bib-0018] Foxe, J.J. , Simpson, G.V. & Ahlfors, S.P. (1998) Parieto‐occipital approximately 10 Hz activity reflects anticipatory state of visual attention mechanisms. NeuroReport, 9, 3929–3933. 9875731 10.1097/00001756-199812010-00030

[ejn13759-bib-0019] Fukuda, K. , Mance, I. & Vogel, E.K. (2015) Alpha power modulation and event‐related slow wave provide dissociable correlates of visual working memory. J. Neurosci., 35, 14009–14016. 26468201 10.1523/JNEUROSCI.5003-14.2015 PMC4604234

[ejn13759-bib-0020] Fukuda, K. , Kang, M.S. & Woodman, G.F. (2016) Distinct neural mechanisms for spatially lateralized and spatially global visual working memory representations. J. Neurophysiol., 116, 1715–1727. 27440249 10.1152/jn.00991.2015 PMC5144708

[ejn13759-bib-0021] Gevins, A. , Smith, M.E. , McEvoy, L. & Yu, D. (1997) High‐resolution EEG mapping of cortical activation related to working memory: effects of task difficulty, type of processing, and practice. Cereb. Cortex, 7, 374–385. 9177767 10.1093/cercor/7.4.374

[ejn13759-bib-0022] Gips, B. , van der Eerden, J.P. & Jensen, O. (2016) A biologically plausible mechanism for neuronal coding organized by the phase of alpha oscillations. Eur. J. Neurosci., 44, 2147–2161. 27320148 10.1111/ejn.13318 PMC5129495

[ejn13759-bib-0023] Griffin, I.C. & Nobre, A.C. (2003) Orienting attention to locations in internal representations. J. Cognitive Neurosci., 15, 1176–1194. 10.1162/089892903322598139 14709235

[ejn13759-bib-0024] Grimault, S. , Robitaille, N. , Grova, C. , Lina, J.M. , Dubarry, A.S. & Jolicoeur, P. (2009) Oscillatory activity in parietal and dorsolateral prefrontal cortex during retention in visual short‐term memory: additive effects of spatial attention and memory load. Hum. Brain Mapp., 30, 3378–3392. 19384891 10.1002/hbm.20759 PMC6870800

[ejn13759-bib-0025] Haegens, S. , Osipova, D. , Oostenveld, R. & Jensen, O. (2010) Somatosensory working memory performance in humans depends on both engagement and disengagement of regions in a distributed network. Hum. Brain Mapp., 31, 26–35. 19569072 10.1002/hbm.20842 PMC6871021

[ejn13759-bib-0026] Haegens, S. , Nácher, V. , Luna, R. , Romo, R. & Jensen, O. (2011a) α‐Oscillations in the monkey sensorimotor network influence discrimination performance by rhythmical inhibition of neuronal spiking. Proc. Natl. Acad. Sci. USA, 108, 19377–19382. 22084106 10.1073/pnas.1117190108 PMC3228466

[ejn13759-bib-0027] Haegens, S. , Händel, B.F. & Jensen, O. (2011b) Top‐down controlled alpha band activity in somatosensory areas determines behavioral performance in a discrimination task. J. Neurosci., 31, 5197–5204. 21471354 10.1523/JNEUROSCI.5199-10.2011 PMC6622699

[ejn13759-bib-0028] Haegens, S. , Barczak, A. , Musacchia, G. , Lipton, M.L. , Mehta, A.D. , Lakatos, P. & Schroeder, C.E. (2015) Laminar profile and physiology of the α rhythm in primary visual, auditory, and somatosensory regions of neocortex. J. Neurosci., 35, 14341–14352. 26490871 10.1523/JNEUROSCI.0600-15.2015 PMC4683691

[ejn13759-bib-0029] Hanslmayr, S. , Staudigl, T. & Fellner, M.C. (2012) Oscillatory power decreases and long‐term memory: the information via desynchronization hypothesis. Front. Hum. Neurosci., 6, 74. 22514527 10.3389/fnhum.2012.00074 PMC3322486

[ejn13759-bib-0030] Hari, R. & Salmelin, R. (1997) Human cortical oscillations: a neuromagnetic view through the skull. Trends Neurosci., 20, 44–49. 9004419 10.1016/S0166-2236(96)10065-5

[ejn13759-bib-0031] Harrison, S.A. & Tong, F. (2009) Decoding reveals the contents of visual working memory in early visual areas. Nature, 458, 632–635. 19225460 10.1038/nature07832 PMC2709809

[ejn13759-bib-0032] Hu, Y. , Hitch, G.J. , Baddeley, A.D. , Zhang, M. & Allen, R.J. (2014) Executive and perceptual attention play different roles in visual working memory: evidence from suffix and strategy effects. J. Exp. Psychol. Human, 40, 1665–1678. 10.1037/a0037163 24933616

[ejn13759-bib-0033] Iemi, L. , Chaumon, M. , Crouzet, S.M. & Busch, N.A. (2017) Spontaneous neural oscillations bias perception by modulating baseline excitability. J. Neurosci., 37, 807–819. 28123017 10.1523/JNEUROSCI.1432-16.2016 PMC6597018

[ejn13759-bib-0034] Ikkai, A. , Blacker, K.J. , Lakshmanan, B.M. , Ewen, J.B. & Courtney, S.M. (2014) Maintenance of relational information in working memory leads to suppression of the sensory cortex. J. Neurophysiol., 112, 1903–1915. 25031260 10.1152/jn.00134.2014 PMC4200014

[ejn13759-bib-0035] Jensen, O. & Mazaheri, A. (2010) Shaping functional architecture by oscillatory alpha activity: gating by inhibition. Front. Hum. Neurosci., 4, 186. 21119777 10.3389/fnhum.2010.00186 PMC2990626

[ejn13759-bib-0036] Jensen, O. , Gelfand, J. , Kounios, J. & Lisman, J.E. (2002) Oscillations in the alpha band (9‐12 Hz) increase with memory load during retention in a short‐term memory task. Cereb. Cortex, 12, 877–882. 12122036 10.1093/cercor/12.8.877

[ejn13759-bib-0037] Jensen, O. , Gips, B. , Bergmann, T.O. & Bonnefond, M. (2014) Temporal coding organized by coupled alpha and gamma oscillations prioritize visual processing. Trends Neurosci., 37, 357–369. 24836381 10.1016/j.tins.2014.04.001

[ejn13759-bib-0038] Johnson, J.S. , Sutterer, D.W. , Acheson, D.J. , Lewis‐Peacock, J.A. & Postle, B.R. (2011) Increased alpha‐band power during the retention of shapes and shape‐location associations in visual short‐term memory. Front. Psychol., 2, 128. 21713012 10.3389/fpsyg.2011.00128 PMC3114253

[ejn13759-bib-0039] Jokisch, D. & Jensen, O. (2007) Modulation of gamma and alpha activity during a working memory task engaging the dorsal or ventral stream. J. Neurosci., 27, 3244–3251. 17376984 10.1523/JNEUROSCI.5399-06.2007 PMC6672464

[ejn13759-bib-0040] Jones, S.R. , Kerr, C.E. , Wan, Q. , Pritchett, D.L. , Hämäläinen, M. & Moore, C.I. (2010) Cued spatial attention drives functionally relevant modulation of the mu rhythm in primary somatosensory cortex. J. Neurosci., 30, 13760–13765. 20943916 10.1523/JNEUROSCI.2969-10.2010 PMC2970512

[ejn13759-bib-0041] Kiyonaga, A. & Egner, T. (2016) Center‐Surround inhibition in working memory. Curr. Biol., 26, 64–68. 26711496 10.1016/j.cub.2015.11.013 PMC4713284

[ejn13759-bib-0042] Klimesch, W. , Doppelmayr, M. , Schwaiger, J. , Auinger, P. & Winkler, T. (1999) ‘Paradoxical’ alpha synchronization in a memory task. Brain Res. Cogn. Brain Res., 7, 493–501. 10076094 10.1016/s0926-6410(98)00056-1

[ejn13759-bib-0043] Klimesch, W. , Sauseng, P. & Hanslmayr, S. (2007) EEG alpha oscillations: the inhibition‐timing hypothesis. Brain Res. Rev., 53, 63–88. 16887192 10.1016/j.brainresrev.2006.06.003

[ejn13759-bib-0044] Kuo, B.C. , Rao, A. , Lepsien, J. & Nobre, A.C. (2009) Searching for targets within the spatial layout of visual short‐term memory. J. Neurosci., 29, 8032–8038. 19553443 10.1523/JNEUROSCI.0952-09.2009 PMC6666054

[ejn13759-bib-0045] Landman, R. , Spekreijse, H. & Lamme, V.A. (2003) Large capacity storage of integrated objects before change blindness. Vision Res., 43, 149–164. 12536137 10.1016/s0042-6989(02)00402-9

[ejn13759-bib-0046] Lange, J. , Oostenveld, R. & Fries, P. (2013) Reduced occipital alpha power indexes enhanced excitability rather than improved visual perception. J. Neurosci., 33, 3212–3220. 23407974 10.1523/JNEUROSCI.3755-12.2013 PMC6619207

[ejn13759-bib-0047] Lee, S.H. , Kravitz, D.J. & Baker, C.I. (2013) Goal‐dependent dissociation of visual and prefrontal cortices during working memory. Nat. Neurosci., 16, 997–999. 23817547 10.1038/nn.3452 PMC3781947

[ejn13759-bib-0048] Lozano‐Soldevilla, D. , ter Huurne, N. , Cools, R. & Jensen, O. (2014) GABAergic modulation of visual gamma and alpha oscillations and its consequences for working memory performance. Curr. Biol., 24, 2878–2887. 25454585 10.1016/j.cub.2014.10.017

[ejn13759-bib-0049] Luck, S.J. & Vogel, E.K. (1997) The capacity of visual working memory for features and conjunctions. Nature, 390, 279–281. 9384378 10.1038/36846

[ejn13759-bib-0050] Makovski, T. , Sussman, R. & Jiang, Y.V. (2008) Orienting attention in visual working memory reduces interference from memory probes. J. Exp. Psychol. Learn., 34, 369–380. 10.1037/0278-7393.34.2.369 18315412

[ejn13759-bib-0051] Medendorp, W.P. , Kramer, G.F. , Jensen, O. , Oostenveld, R. , Schoffelen, J.M. & Fries, P. (2007) Oscillatory activity in human parietal and occipital cortex shows hemispheric lateralization and memory effects in a delayed double‐step saccade task. Cereb. Cortex, 17, 2364–2374. 17190968 10.1093/cercor/bhl145

[ejn13759-bib-0052] Mok, R.M. , Myers, N.E. , Wallis, G. & Nobre, A.C. (2016) Behavioral and neural markers of flexible attention over working memory in aging. Cereb. Cortex, 26, 1831–1842. 26865653 10.1093/cercor/bhw011 PMC4785959

[ejn13759-bib-0053] Myers, N.E. , Stokes, M.G. , Walther, L. & Nobre, A.C. (2014) Oscillatory brain state predicts variability in working memory. J. Neurosci., 34, 7735–7743. 24899697 10.1523/JNEUROSCI.4741-13.2014 PMC4044240

[ejn13759-bib-0054] Myers, N.E. , Walther, L. , Wallis, G. , Stokes, M.G. & Nobre, A.C. (2015) Temporal dynamics of attention during encoding versus maintenance of working memory: complementary views from event‐related potentials and alpha‐band oscillations. J. Cognitive Neurosci., 27, 492–508. 10.1162/jocn_a_00727 PMC4678590 25244118

[ejn13759-bib-0055] Myers, N.E. , Stokes, M.G. & Nobre, A.C. (2017) Prioritizing information during working memory: beyond sustained internal attention. Trends Cogn. Sci., 21, 449–461. 28454719 10.1016/j.tics.2017.03.010 PMC7220802

[ejn13759-bib-0056] Niklaus, M. , Nobre, A.C. & van Ede, F. (2017) Feature‐based attentional weighting and spreading in visual working memory. Sci. Rep., 7, 42384. 28233830 10.1038/srep42384 PMC5324041

[ejn13759-bib-0057] Oberauer, K. (2002) Access to information in working memory: exploring the focus of attention. J. Exp. Psychol. Learn., 28, 411–421. 12018494

[ejn13759-bib-0058] Obleser, J. , Wöstmann, M. , Hellbernd, N. , Wilsch, A. & Maess, B. (2012) Adverse listening conditions and memory load drive a common oscillatory network. J. Neurosci., 32, 12376–12383. 22956828 10.1523/JNEUROSCI.4908-11.2012 PMC6621258

[ejn13759-bib-0059] Palva, S. & Palva, J.M. (2007) New vistas for alpha‐frequency band oscillations. Trends Neurosci., 30, 150–158. 17307258 10.1016/j.tins.2007.02.001

[ejn13759-bib-0060] Palva, J.M. , Monto, S. , Kulashekhar, S. & Palva, S. (2010) Neuronal synchrony reveals working memory networks and predicts individual memory capacity. Proc. Natl. Acad. Sci. USA, 107, 7580–7585. 20368447 10.1073/pnas.0913113107 PMC2867688

[ejn13759-bib-0061] Pasternak, T. & Greenlee, M.W. (2005) Working memory in primate sensory systems. Nat. Rev. Neurosci., 6, 97–107. 15654324 10.1038/nrn1603

[ejn13759-bib-0062] Payne, L. & Sekuler, R. (2014) The importance of ignoring: alpha oscillations protect selectivity. Curr. Dir. Psychol. Sci., 23, 171–177. 25530685 10.1177/0963721414529145 PMC4266987

[ejn13759-bib-0063] Poch, C. , Campo, P. & Barnes, G.R. (2014) Modulation of alpha and gamma oscillations related to retrospectively orienting attention within working memory. Eur. J. Neurosci., 40, 2399–2405. 24750388 10.1111/ejn.12589 PMC4215597

[ejn13759-bib-0064] Poliakov, E. , Stokes, M.G. , Woolrich, M.W. , Mantini, D. & Astle, D.E. (2014) Modulation of alpha power at encoding and retrieval tracks the precision of visual short‐term memory. J. Neurophysiol., 112, 2939–2945. 25210151 10.1152/jn.00051.2014 PMC4254886

[ejn13759-bib-0065] Rademaker, R.L. , Bloem, I.M. , De Weerd, P. & Sack, A.T. (2015) The impact of interference on short‐term memory for visual orientation. J. Exp. Psychol. Human, 41, 1650–1665. 10.1037/xhp0000110 26371383

[ejn13759-bib-0066] Roberts, B.M. , Hsieh, L.T. & Ranganath, C. (2013) Oscillatory activity during maintenance of spatial and temporal information in working memory. Neuropsychologia, 51, 349–357. 23084981 10.1016/j.neuropsychologia.2012.10.009 PMC3546228

[ejn13759-bib-0067] Roux, F. & Uhlhaas, P.J. (2014) Working memory and neural oscillations: α‐γ versus θ‐γ codes for distinct WM information? Trends Cogn. Sci., 18, 16–25. 24268290 10.1016/j.tics.2013.10.010

[ejn13759-bib-0068] Sauseng, P. , Klimesch, W. , Heise, K.F. , Gruber, W.R. , Holz, E. , Karim, A.A. , Glennon, M. , Gerloff, C. *et al* (2009) Brain oscillatory substrates of visual short‐term memory capacity. Curr. Biol., 19, 1846–1852. 19913428 10.1016/j.cub.2009.08.062

[ejn13759-bib-0069] Schneider, D. , Mertes, C. & Wascher, E. (2015) On the fate of non‐cued mental representations in visuo‐spatial working memory: Evidence by a retro‐cuing paradigm. Behav. Brain Res., 293, 114–124. 26196953 10.1016/j.bbr.2015.07.034

[ejn13759-bib-0070] Serences, J.T. , Ester, E.F. , Vogel, E.K. & Awh, E. (2009) Stimulus‐specific delay activity in human primary visual cortex. Psychol. Sci., 20, 207–214. 19170936 10.1111/j.1467-9280.2009.02276.x PMC2875116

[ejn13759-bib-0071] Siebenhühner, F. , Wang, S.H. , Palva, J.M. & Palva, S. (2016) Cross‐frequency synchronization connects networks of fast and slow oscillations during visual working memory maintenance. Elife, 5, e13451. 27669146 10.7554/eLife.13451 PMC5070951

[ejn13759-bib-0072] Snyder, A.C. & Foxe, J.J. (2010) Anticipatory attentional suppression of visual features indexed by oscillatory alpha‐band power increases: a high‐density electrical mapping study. J. Neurosci., 30, 4024–4032. 20237273 10.1523/JNEUROSCI.5684-09.2010 PMC2908241

[ejn13759-bib-0073] Souza, A.S. & Oberauer, K. (2016) In search of the focus of attention in working memory: 13 years of the retro‐cue effect. Atten. Percept. Psycho., 78, 1839–1860. 10.3758/s13414-016-1108-5 27098647

[ejn13759-bib-0074] Souza, A.S. , Rerko, L. & Oberauer, K. (2016) Getting more from visual working memory: retro‐cues enhance retrieval and protect from visual interference. J. Exp. Psychol. Human, 42, 890–910. 10.1037/xhp0000192 26752731

[ejn13759-bib-0075] Spitzer, B. & Blankenburg, F. (2011) Stimulus‐dependent EEG activity reflects internal updating of tactile working memory in humans. Proc. Natl. Acad. Sci. USA, 108, 8444–8449. 21536865 10.1073/pnas.1104189108 PMC3100957

[ejn13759-bib-0076] Spitzer, B. & Blankenburg, F. (2012) Supramodal parametric working memory processing in humans. J. Neurosci., 32, 3287–3295. 22399750 10.1523/JNEUROSCI.5280-11.2012 PMC6621033

[ejn13759-bib-0077] Sreenivasan, K.K. & Jha, A.P. (2007) Selective attention supports working memory maintenance by modulating perceptual processing of distractors. J. Cognitive Neurosci., 19, 32–41. 10.1162/jocn.2007.19.1.32 17214561

[ejn13759-bib-0078] Sreenivasan, K.K. , Curtis, C.E. & D'Esposito, M. (2014) Revisiting the role of persistent neural activity during working memory. Trends Cogn. Sci., 18, 82–89. 24439529 10.1016/j.tics.2013.12.001 PMC3964018

[ejn13759-bib-0079] Staresina, B.P. , Michelmann, S. , Bonnefond, M. , Jensen, O. , Axmacher, N. & Fell, J. (2016) Hippocampal pattern completion is linked to gamma power increases and alpha power decreases during recollection. Elife, 5, e17397. 27508355 10.7554/eLife.17397 PMC4980114

[ejn13759-bib-0080] Thut, G. & Miniussi, C. (2009) New insights into rhythmic brain activity from TMS‐EEG studies. Trends Cogn. Sci., 13, 182–189. 19286414 10.1016/j.tics.2009.01.004

[ejn13759-bib-0081] Thut, G. , Nietzel, A. , Brandt, S.A. & Pascual‐Leone, A. (2006) Alpha‐band electroencephalographic activity over occipital cortex indexes visuospatial attention bias and predicts visual target detection. J. Neurosci., 26, 9494–9502. 16971533 10.1523/JNEUROSCI.0875-06.2006 PMC6674607

[ejn13759-bib-0082] Thut, G. , Miniussi, C. & Gross, J. (2012) The functional importance of rhythmic activity in the brain. Curr. Biol., 22, R658–R663. 22917517 10.1016/j.cub.2012.06.061

[ejn13759-bib-0083] Tuladhar, A.M. , ter Huurne, N. , Schoffelen, J.M. , Maris, E. , Oostenveld, R. & Jensen, O. (2007) Parieto‐occipital sources account for the increase in alpha activity with working memory load. Hum. Brain Mapp., 28, 785–792. 17266103 10.1002/hbm.20306 PMC6871495

[ejn13759-bib-0084] Vogel, E.K. & Machizawa, M.G. (2004) Neural activity predicts individual differences in visual working memory capacity. Nature, 428, 748–751. 15085132 10.1038/nature02447

[ejn13759-bib-0085] de Vries, I.E.J. , van Driel, J. & Olivers, C.N.L. (2017) Posterior α EEG dynamics dissociate current from future goals in working memory‐guided visual search. J. Neurosci., 37, 1591–1603. 28069918 10.1523/JNEUROSCI.2945-16.2016 PMC5299573

[ejn13759-bib-0086] Waldhauser, G.T. , Braun, V. & Hanslmayr, S. (2016) Episodic memory retrieval functionally relies on very rapid reactivation of sensory information. J. Neurosci., 36, 251–260. 26740665 10.1523/JNEUROSCI.2101-15.2016 PMC6601789

[ejn13759-bib-0087] Wallis, G. , Stokes, M. , Cousijn, H. , Woolrich, M. & Nobre, A.C. (2015) Frontoparietal and cingulo‐opercular networks play dissociable roles in control of working memory. J. Cognitive Neurosci., 27, 2019–2034. 10.1162/jocn_a_00838 26042457

[ejn13759-bib-0088] Williams, M. , Pouget, P. , Boucher, L. & Woodman, G.F. (2013) Visual‐spatial attention aids the maintenance of object representations in visual working memory. Mem. Cognition, 41, 698–715. 10.3758/s13421-013-0296-7 PMC3655125 23371773

[ejn13759-bib-0089] Wolff, M.J. , Jochim, J. , Akyürek, E.G. & Stokes, M.G. (2017) Dynamic hidden states underlying working‐memory‐guided behavior. Nat. Neurosci., 20, 864–871. 28414333 10.1038/nn.4546 PMC5446784

[ejn13759-bib-0090] Worden, M.S. , Foxe, J.J. , Wang, N. & Simpson, G.V. (2000) Anticipatory biasing of visuospatial attention indexed by retinotopically specific alpha‐band electroencephalography increases overoccipital cortex. J. Neurosci., 20, RC63. 10704517 10.1523/JNEUROSCI.20-06-j0002.2000 PMC6772495

[ejn13759-bib-0091] Wöstmann, M. , Lim, S.J. & Obleser, J. (2017) The human neural alpha response to speech is a proxy of attentional control. Cereb. Cortex, 27, 3307–3317. 28334352 10.1093/cercor/bhx074

[ejn13759-bib-0092] Ye, C. , Hu, Z. , Ristaniemi, T. , Gendron, M. & Liu, Q. (2016) Retro‐dimension‐cue benefit in visual working memory. Sci. Rep., 6, 35573. 27774983 10.1038/srep35573 PMC5075867

[ejn13759-bib-0093] Zohary, E. , Shadlen, M.N. & Newsome, W.T. (1994) Correlated neuronal discharge rate and its implications for psychophysical performance. Nature, 370, 140–143. 8022482 10.1038/370140a0

[ejn13759-bib-0094] Zokaei, N. , Ning, S. , Manohar, S. , Feredoes, E. & Husain, M. (2014) Flexibility of representational states in working memory. Front. Hum. Neurosci., 8, 853. 25414654 10.3389/fnhum.2014.00853 PMC4222142

