# Supplementary material for: Mnemonic and attentional roles for states of attenuated alpha oscillations in perceptual working memory: a review
Source: Eur J Neurosci. 2017 Nov 22;48(7):2509–15. doi: 10.1111/ejn.13759 (PMC6220786; doi:10.1111/ejn.13759)
Supplement: Supplementary file 1 [file EJN-48-2509-s001.pdf]

EJN European Journal of Neuroscience FENS Federation of European Neuroscience Societies

Mnemonic and attentional roles for states of attenuated alpha oscillations in perceptual working memory: a review

Freek van Ede

Review timeline:

Submission date:	27 June 2017
Editorial Decision:	05 September 2017
Revision received:	07 September 2017
Accepted:	19 October 2017

Editor: Ali Mazaheri

1st Editorial Decision 05 September 2017

Dear Dr. van Ede,

Your review paper has now been thoroughly reviewed by two external reviewers, by our Guest Section Editor Dr. Ali Mazaheri, and by ourselves. All concerned feel that the paper is both timely and will be of significant interest to the readers of the special issue. Nonetheless, the reviewers have raised a number of issues that should be dealt with in a revision before we can proceed. There are calls for consideration of additional literature, for you to make clear what the novel contributions of the review are, for a more mechanistic perspective, and for ways to improve the clarity and flow of your arguments.

If you are able to respond fully to the points raised, we would be pleased to receive a revision of your paper within 30 days.

Thank you for submitting your work to EJN.

Kind regards,

John Foxe & Paul Bolam
co-Editors in Chief, EJN

Reviews:

Reviewer: 1 (Sam Doesburg, Simon Fraser University, Canada)

Comments to the Author
The authors review the role of alpha oscillations in working memory and review their purported mnemonic properties. Specifically they contrast visual and nonvisual content in terms of its impact on alpha oscillations (particularly over posterior cortex), as well as arguing against several alternative explanations which are in competition with mnemonic accounts. Overall this is a fairly comprehensive and well reasoned position paper although I do feel there are a few things which could be addressed.

Much has been written on the role of alpha oscillations in neural function and cognition generally, and in working memory specifically. Given this I think the manuscript could be improved by explaining what is novel about the current contribution – what sets it apart from prior works considering the role of alpha oscillations in WM processing.

There are other papers which show attenuation of alpha oscillations during visual WM which should be cited given this is the focus of this paper.

The authors do a good job of contrasting how various cognitive accounts of modulation of alpha oscillations in the context of WM and related processing. I think what is missing is how this might fit into a more mechanistic view of alpha oscillations. In recent years there has been considerable work on how such oscillations likely arise through synchronized dendritic currents reflecting temporal windows of

excitation/inhibition. In this view these my not be ‘process specific’ in the cognitive sense, but pertain to a more fundamental and mechanistic set of processes that underlie multiple processes. I think this perspective should be addressed in some detail. This is not necessarily at odds with the mnemonic account but may enrich the discussion somewhat.

Reviewer: 2 (Kartik Sreenivasan, New York University Abu Dhabi, United Arab Emirates)

Comments to the Author
Summary:
This review considers evidence for modulations of alpha power during working memory and argues that, at least in the case of ‘perceptual’ WM, alpha power is reduced during WM and this reduction serves a mnemonic role. The author then considers alternative roles for alpha attenuation during WM.

General Comments:
There has been an increasing interest in the relationship between alpha power and WM, so this work is quite timely. It also makes a nice attempt to reconcile important discrepancies in the literature by distinguishing perceptual and non-perceptual WM (although I have some reservations about this – see below). Overall, I find the argument to be convincing and believe that this work will be of interest to researchers interested in WM and/or the function of alpha oscillations. Below I outline specific concerns and suggestions.

Major Comments:
(1) The main argument that alpha serves a mnemonic function has two parts. First, previous work showing alpha enhancement usually used ‘non-perceptual’ WM and, therefore, may reflect sensory disengagement to protect from interference. Second, studies employing ‘perceptual’ WM demonstrate alpha attenuation in the relevant sensory regions. There are two issues with this argument:
A. While it’s compelling to reinterpret previous work as showing sensory disengagement for non-sensory WM, the evidence that alpha in these studies (e.g., Jensen et al., 2002) was generated in sensory regions is scant. The author seems to acknowledge as much, referring to posterior alpha modulations as “putatively visual”. However, without direct evidence that alpha enhancement during non-visual WM is generated in visual areas, this part of the argument is a bit speculative. If such evidence exists, it should be stated more clearly.
B. How does one reconcile alpha attenuation (i.e., enhanced processing) in relevant sensory regions during perceptual WM with findings that (i) perceptually *similar* distractors produce more behavioral interference during WM than dissimilar distractors during perceptual WM (Rademaker et al., 2015; Chumbley et al., 2008), and (ii) similar stimuli show greater behavioral (Kiyonaga et al., 2015) and neural evidence (Sreenivasan et al., 2007) for suppression during perceptual WM? Put another way, isn’t it more important to disengage from sensory processing of a Gabor oriented at 45 degrees when one is maintaining at Gabor oriented at 70 degrees as opposed to when one is maintaining a list of letters? Thus, the benefit of generally enhanced sensory processing during perceptual WM to WM performance is unclear.

(2) I’m not clear how the third alternative account (retrocue processing) is different than the second alternative (lingering sensory processing). If there is an important difference between processing of items at encoding and processing of the retrocue, this should be explicitly stated. It seems to me that both involve sensory processing as well as selection, be it selection of external information or internal information. Further, the other four alternatives are presented as operations that alpha modulations may be representing, whereas this one is presented narrowly as processing of a specific stimulus in a particular task. If it’s important to make the case that this is different than lingering sensory processing, then it should be reframed and described in terms of a more general alternative function.

(3) Multivariate analyses of alpha power are not considered thoroughly enough. For example, Foster et al., 2016 uses an inverted encoding model to show that the pattern of alpha power across the scalp is spatially tuned for the maintained location. I believe this paper (and other similar papers) makes the strongest case for alpha involvement in WM, as it shows that information in WM can be decoded from alpha power. The author incorrectly equates this encoding analysis with pattern classification and dismisses both as being too black-boxy to interpret; however, encoding models make explicit assumptions about how information is encoded in brain activity, allowing an interpretation that is more closely tied to brain physiology. The interpretation of these results in the present framework of alpha enhancement and attenuation is certainly more challenging because decoding is driven by a (potentially complex) pattern of alpha enhancement and attenuation. However, these results still provide strong positive evidence that alpha oscillations encode WM information, and consequently warrant further discussion.

Minor Comments:
(4) The flow of arguments in the Introduction starts getting a bit confusing in the 4th paragraph. Up until this point, the reader is presented with evidence for alpha *enhancement* during WM. Then, in the fourth paragraph, there are two key points: 1. That alpha enhancement may reflect sensory disengagement only in cases where sensory information is not being maintained; 2. That alpha *attenuation* may serve a mnemonic function. The last sentence of this paragraph suggests only the 2nd point will be reviewed in this

paper, although we return to the 1st point in the next section. It seems that the first point is worth the readers' attention, particularly as it returns later in the paper. With regards to the second point, there is no mention of evidence for alpha attenuation until the next section of the paper, so it's a bit confusing to hear about it in this way in the fourth paragraph of the Introduction.

(5) Page 5, in the heading 'Mnemonic retention vs. lingering of sensory encoding': "…appear to sustain throughout encoding intervals of three or more seconds…" – shouldn't this be 'retention' and not 'encoding'? If not, then I'm not sure this argument makes sense.

(6) Page 5, in the heading 'Mnemonic retention v. probe anticipation': "…may thus be account for…" should be "may thus account for".

Authors' Response 07 September 2017

Point-by-point replies

Reviewer 1
The authors review the role of alpha oscillations in working memory and review their purported mnemonic properties. Specifically they contrast visual and nonvisual content in terms of its impact on alpha oscillations (particularly over posterior cortex), as well as arguing against several alternative explanations which are in competition with mnemonic accounts. Overall this is a fairly comprehensive and well reasoned position paper although I do feel there are a few things which could be addressed.

Much has been written on the role of alpha oscillations in neural function and cognition generally, and in working memory specifically. Given this I think the manuscript could be improved by explaining what is novel about the current contribution – what sets it apart from prior works considering the role of alpha oscillations in WM processing.

Thank you for pointing this out. I have now start the last paragraph of the introduction with the following:

> "Building on earlier reviews on the role of alpha oscillations in WM that focused and converged on a protective (disengaging) role for states of amplified alpha oscillations during WM (Roux and Uhlhaas, 2014; Payne and Sekuler, 2014), I here review recent evidence for this complementary mnemonic role for states of attenuated alpha oscillations during perceptual WM. […]"

In addition, I thereafter state that:

> "In addition, I discuss recent evidence that such attenuated alpha states may not only support WM retention, but may also support attentional prioritisation within WM and govern item accessibility."

And that:

> "I finally consider (and, where possible, counter) four alternative interpretations of the available data that challenge a strictly mnemonic interpretation, and highlight key implications of the reviewed work."

I hope that listing these aims helps to appreciate the novel contributions of the current review: (1) focusing on states of alpha attenuation during perceptual working memory, and embedding the literature on alpha with recent "sensory recruitment" models of working memory (which are referred to throughout), (2) also reviewing literature on attentional prioritisation within working memory, and (3) also considering multiple alternative accounts.

There are other papers which show attenuation of alpha oscillations during visual WM which should be cited given this is the focus of this paper.

Thank you. I agree and have now added the following:

> "[…] For more demonstrations of alpha attenuation during WM see also: Medendorp et al., 2007; Fukuda et al., 2015; Erickson et al., 2017".

(These are the main papers that I can think of. It seems to me that most other papers either involved a contrast between two WM conditions (leaving the direction of the effect unresolved, as pointed out in the review) or involved a power increase. If the reviewer is aware of particular studies that I may well have overlooked, I am of course happy to be informed on this, and to include these as well).

The authors do a good job of contrasting how various cognitive accounts of modulation of alpha oscillations in the context of WM and related processing. I think what is missing is how this might fit into a more mechanistic view of alpha oscillations. In recent years there has been considerable work on how such oscillations likely arise through synchronized dendritic currents reflecting temporal windows of excitation/inhibition. In this view these my not be 'process specific' in the cognitive sense, but pertain to a more fundamental and mechanistic set of processes that underlie multiple processes. I think this perspective should be addressed in some detail. This is not necessarily at odds with the mnemonic account but may enrich the discussion somewhat.

Thank you for bringing this important point forward. I agree and now elaborate on this in the following way:

> "A key open question regards the precise nature of the neural computations associated with (or enabled by) states of attenuated alpha oscillations (as well as their means of carrying over into measurable modulations in extracranial M/EEG). While the answer to this important question remains largely unknown, it is worth pointing out that these neural computations are unlikely to carry only a mnemonic function. Indeed, it is well known that sensory processing also attenuates alpha oscillations, and that such attenuation can also be instantiated during the mere anticipation of upcoming processing demands (e.g., Foxe et al., 1998; Worden et al., 2000; Thut et al., 2006; Jones et al., 2010; Haegens et al., 2011b; van Ede et al., 2011; 2012). It is thus likely that, rather than "coding" for the items in WM themselves, such states enable neural computations that facilitate information processing, be it in the context of perception, action, working memory, and so on. Influential models posit that alpha oscillations may be the consequence of rhythmic pulses of inhibition in which individual cycles contain only relatively short "windows of opportunity" (when inhibition dies off) for neurons to fire and exchange information (Klimesch et al., 2007; Jensen and Mazaheri, 2010; Jensen et al., 2014; Gips et al., 2016). If so, their attenuation may reflect reduced inhibitory pulsing, creating longer windows of opportunity and enhancing (the capacity for) information processing and transmission. Another, potentially complementary, possibility is that attenuated alpha states are associated with the decorrelation (desynchronisation) of neuronal firing rates (potentially through the segregation into multiple alpha sub-networks at a finer spatial scale), which may increase the coding capacity of the corresponding neuronal population (Zohary et al., 1994; Hanslmayr et al., 2012). Clearly more work is needed to investigate these possibilities and how they relate to mnemonic as well as other cognitive processes and neural computations."

In addition, to provide more structure, I have now broken down the text in the "Implications and future directions" section (essentially the discussion) into the following two sub sections: "alpha oscillations and cognition" and "working memory and distractibility".

Reviewer 2

This review considers evidence for modulations of alpha power during working memory and argues that, at least in the case of 'perceptual' WM, alpha power is reduced during WM and this reduction serves a mnemonic role. The author then considers alternative roles for alpha attenuation during WM.

General Comments

There has been an increasing interest in the relationship between alpha power and WM, so this work is quite timely. It also makes a nice attempt to reconcile important discrepancies in the literature by distinguishing perceptual and non-perceptual WM (although I have some reservations about this – see below). Overall, I find the argument to be convincing and believe that this work will be of interest to researchers interested in WM and/or the function of alpha oscillations. Below I outline specific concerns and suggestions.

Major Comments
(1) The main argument that alpha serves a mnemonic function has two parts. First, previous work showing alpha enhancement usually used ‘non-perceptual’ WM and, therefore, may reflect sensory disengagement to protect from interference. Second, studies employing ‘perceptual’ WM demonstrate alpha attenuation in the relevant sensory regions. There are two issues with this argument:

A. While it’s compelling to reinterpret previous work as showing sensory disengagement for non-sensory WM, the evidence that alpha in these studies (e.g., Jensen et al., 2002) was generated in sensory regions is scant. The author seems to acknowledge as much, referring to posterior alpha modulations as “putatively visual”. However, without direct evidence that alpha enhancement during non-visual WM is generated in visual areas, this part of the argument is a bit speculative. If such evidence exists, it should be stated more clearly.

Thank you for bringing this up. While it is true that the localisation of the alpha amplification reported in Jensen et al. (2002) was not pin-pointed to particular anatomical (visual) areas, later work has demonstrated that the main sources of these alpha amplifications involve occipital areas. I now state this explicitly:

> “Indeed, amplification of posterior alpha oscillations appears particularly prominent in tasks where the content of WM is verbal, or somatosensory (Jensen et al., 2002; Haegens et al., 2010; Bonnefond and Jensen, 2012; see also Gevins et al., 1997; but see Johnson et al., 2011), but where the sources of this amplification localise to visual brain areas (Tuladhar et al., 2007; Haegens et al., 2010; Bonnefond and Jensen, 2012).”

B. How does one reconcile alpha attenuation (i.e., enhanced processing) in relevant sensory regions during perceptual WM with findings that (i) perceptually *similar* distractors produce more behavioral interference during WM than dissimilar distractors during perceptual WM (Rademaker et al., 2015; Chumbley et al., 2008), and (ii) similar stimuli show greater behavioral (Kiyonaga et al., 2015) and neural evidence (Sreenivasan et al., 2007) for suppression during perceptual WM? Put another way, isn’t it more important to disengage from sensory processing of a Gabor oriented at 45 degrees when one is maintaining at Gabor oriented at 70 degrees as opposed to when one is maintaining a list of letters? Thus, the benefit of generally enhanced sensory processing during perceptual WM to WM performance is unclear.

Thank you for bringing this important and relevant work to my attention. I fully agree that it is more urgent to suppress similar (more interfering) sensory inputs that are (re)presented in the same modality, location, or feature-dimension as the memoranda. However, while this may be more urgent, this does not also mean that this can also easily be accomplished. In fact, as I have tried to argue in the “Implications and future directions” section, the attenuation of alpha (or sensory recruitment in general) for perceptual WM may be beneficial for preserving the high perceptual resolution of the memoranda, but may come at the cost of increased susceptibility to distraction of perceptually similar items. I believe the results of Sreenivasan, Rademaker and Chumbley nicely tie in with this argument and I have added these references accordingly:

> “One implication of the reviewed work may be that the high perceptual resolution gained from retaining items in early sensory areas (putatively through attenuated alpha states) inherently comes at the cost of increased susceptibility to sensory interference from similar material (in line with e.g., Sreenivasan and Jha, 2007; Chumbley et al., 2008; Rademaker et al., 2015).”

In addition, I now continue with the following in response to the second set of literature referred to by the reviewer:

> “[…] At the same time, the fact that distractors that are perceptually more similar to retained WM representations lead to more interference does not mean that such distraction cannot be overcome at all. Indeed, there is evidence that perceptually similar items may sometimes even receive more inhibition than less similar items (Sreenivasan and Jha, 2007; Kiyonaga and Egner, 2016). Whether and how alpha modulations during retention can support this remains to be investigated, and will depend a lot on the degree of spatial and feature specificity with which alpha can be modulated within mnemonically relevant sensory areas.”

(2) I'm not clear how the third alternative account (retrocue processing) is different than the second alternative (lingering sensory processing). If there is an important difference between processing of items at encoding and processing of the retrocue, this should be explicitly stated. It seems to me that both involve sensory processing as well as selection, be it selection of external information or internal information. Further, the other four alternatives are presented as operations that alpha modulations may be representing, whereas this one is presented narrowly as processing of a specific stimulus in a particular task. If it's important to make the case that this is different than lingering sensory processing, then it should be reframed and described in terms of a more general alternative function.

Thank you for pointing this out. I agree that these two points refer to qualitatively similar alternative accounts related to sensory (as opposed to mnemonic) processing. The reason I had separated them is that the retro-cue processing alternative is only relevant for a particular set of studies (those dealing with attentional prioritisation), whereas the lingering of encoding alternative is relevant for all. Based on the reviewer's comment, I have now grouped the two sections under a single header (and now refer throughout to the four instead of the five alternative accounts).

(3) Multivariate analyses of alpha power are not considered thoroughly enough. For example, Foster et al., 2016 uses an inverted encoding model to show that the pattern of alpha power across the scalp is spatially tuned for the maintained location. I believe this paper (and other similar papers) makes the strongest case for alpha involvement in WM, as it shows that information in WM can be decoded from alpha power. The author incorrectly equates this encoding analysis with pattern classification and dismisses both as being too black-boxy to interpret; however, encoding models make explicit assumptions about how information is encoded in brain activity, allowing an interpretation that is more closely tied to brain physiology. The interpretation of these results in the present framework of alpha enhancement and attenuation is certainly more challenging because decoding is driven by a (potentially complex) pattern of alpha enhancement and attenuation. However, these results still provide strong positive evidence that alpha oscillations encode WM information, and consequently warrant further discussion.

I agree and have now rewritten this to the following:

> "Several recent studies have further demonstrated a link between alpha oscillations during retention and the content-specific identities of WM representations using sophisticated forward encoding modelling of visual stimulus features such as visual orientation (e.g. Foster et al., 2015; Fukuda et al., 2016). This work too argues for a mnemonic role for alpha oscillations during perceptual WM and shows that this role extends beyond the modality and spatial location of the mnemonic items to also include their feature-specific identity. It remains to be evaluated, however, precisely what aspects of the alpha oscillations (spatial patterns of attenuation, amplification, or both, as well as orthogonal aspects such as changes in peak frequency) contribute to the reconstruction of the memorised stimulus features."

Minor Comments

(4) The flow of arguments in the Introduction starts getting a bit confusing in the 4th paragraph. Up until this point, the reader is presented with evidence for alpha *enhancement* during WM. Then, in the fourth paragraph, there are two key points: 1. That alpha enhancement may reflect sensory disengagement only in cases where sensory information is not being maintained; 2. That alpha *attenuation* may serve a mnemonic function. The last sentence of this paragraph suggests only the 2nd point will be reviewed in this paper, although we return to the 1st point in the next section. It seems that the first point is worth the readers' attention, particularly as it returns later in the paper. With regards to the second point, there is no mention of evidence for alpha attenuation until the next section of the paper, so it's a bit confusing to hear about it in this way in the fourth paragraph of the Introduction.

Thank you. I no longer state that review is only about second point. Furthermore, in response to a concern from reviewer 1, I have also reformulated the last paragraph of the introduction that should hopefully also satisfy this comment.

(5) Page 5, in the heading 'Mnemonic retention vs. lingering of sensory encoding': "…appear to sustain

throughout encoding intervals of three or more seconds…" – shouldn't this be 'retention' and not 'encoding'? If not, then I'm not sure this argument makes sense.

This should indeed be retention; thank you for spotting this.

(6) Page 5, in the heading 'Mnemonic retention v. probe anticipation': "…may thus be account for…" should be "may thus account for".

Thank you; I have corrected this.

References

Bonnefond M, Jensen O (2012) Alpha oscillations serve to protect working memory maintenance against anticipated distracters. Curr Biol 22:1969-1974.

Chumbley JR, Dolan RJ, Friston KJ (2008) Attractor models of working memory and their modulation by reward. Biol Cybern 98:11–18.

Erickson, MA, Albrecht MA, Robinson B, Luck SJ, Gold JM (2017) Impaired suppression of delay-period alpha and beta is associated with impaired working memory in schizophrenia. Biol Psychiatry 2:272-279.

Fukuda K, Mance I, Vogel EK (2015) Alpha power modulation and event-related slow wave provide dissociable correlates of visual working memory. J Neurosci 35:14009-14016.

Gips B, van der Eerden JP, Jensen O (2016) A biologically plausible mechanism for neuronal coding organized by the phase of alpha oscillations. Eur J Neurosci 44:2147-2161.

Jensen O, Gips B, Bergmann TO, Bonnefond M (2014) Temporal coding organized by coupled alpha and gamma oscillations prioritize visual processing. Trends Neurosci 37:357-369.

Kiyonaga A, Egner T (2016) Center-Surround inhibition in working memory. Curr Biol 26:64-68.

Medendorp WP, Kramer GF, Jensen O, Oostenveld R, Schoffelen JM, Fries P (2007) Oscillatory activity in human parietal and occipital cortex shows hemispheric lateralization and memory effects in a delayed double-step saccade task. Cereb Cortex 17:2364-2374.

Rademaker RL, Bloem IM, De Weerd P, Sack AT (2015) The impact of interference on short-term memory for visual orientation. J Exp Psychol Hum Percept Perform 41:1650-1665.

Sreenivasan KK, Jha AP (2007) Selective attention supports working memory maintenance by modulating perceptual processing of distractors. J Cogn Neurosci 19:32-41.

Tuladhar AM, ter Huurne N, Schoffelen JM, Maris E, Oostenveld R, Jensen O (2007) Parieto-occipital sources account for the increase in alpha activity with working memory load. Hum Brain Mapp 28:785-792.
